# Investigating the Relationship between Job Burnout and Job Satisfaction among Chinese Generalist Teachers in Rural Primary Schools: A Serial Mediation Model

**DOI:** 10.3390/ijerph192114427

**Published:** 2022-11-03

**Authors:** Wei Chen, Shuyi Zhou, Wen Zheng, Shiyong Wu

**Affiliations:** 1 School of Education, Huizhou University, Huizhou 516000, China; 2 College of Education, Zhejiang University, Hangzhou 310000, China; 3 South China Vocational Education Research Centre, South China Normal University, Shanwei 516600, China

**Keywords:** generalist teacher, mental health, occupational health, perceived organizational support, work engagement, work-related well-being

## Abstract

Background: Job burnout has become a widespread phenomenon in school settings. However, little is known about the mental health condition of the growing cohort of generalist teachers in rural primary schools. Drawing on the job demands–resource model and social exchange theory, this study examines the correlation between job burnout and job satisfaction through perceived organizational support and work engagement. Methods: We recruited 639 Chinese generalist teachers in rural primary schools as the study subjects and developed a serial mediation model to assess the hypothesized construct. The data acquired online via Wenjuanxing Software were confirmed as valid and analyzed with SPSS and SmartPLS. Results: The participants scored above the median in perceived organizational support, work engagement, and job satisfaction, and had scores close to the median for job burnout. Statistically significant differences among the investigated variables were found in gender, age, experience, and degree. Job burnout was negatively predictive of perceived organizational support, work engagement, and job satisfaction (each *p* = 0.000). Perceived organizational support mediated the association between job burnout and work engagement (*p* = 0.000) and the connection between job burnout and job satisfaction (*p* = 0.000), while work engagement mediated the association between job burnout and job satisfaction (*p* = 0.000) and the association between perceived organizational support and job satisfaction (*p* = 0.000). Conclusions: Perceived organizational support and work engagement as two sequential mediators buffered the detrimental impact of job burnout on job satisfaction among Chinese generalist teachers in rural primary schools. Targeted strategies should be implemented to diminish generalist teachers’ feelings of burnout, being unsupported by organizations, disengagement at work, and dissatisfaction with the job.

## 1. Introduction

Job burnout is widely described as “a psychological syndrome of emotional exhaustion, depersonalization, and reduced professional efficacy” [1,2]. Factors contributing to job burnout can be summarized into two interactional forms: organizational and individual [3]. Regarding the organizational factors, employees’ perceived organizational support, mainly from leaders and fellows, has been significantly associated with their feelings of job burnout [4,5]. Concerning the individual factors, employees’ attitudes, and sentiments towards work conditions, such as work engagement and job satisfaction, have also been closely connected with the occurrence of job burnout [6,7].

Perceived organizational support reflects an employee’s viewpoint that their contributions are valued and their benefits are concerned for by their employers [8]. Some burnout studies have disclosed that perceived organizational support contributes to employees’ job burnout in diverse work contexts [9,10,11]. Conversely, long-term burnout can cause an employee to feel unsupported, uncared for, and unappreciated by the organization, and could even result in a decrease in loyalty [12]. Work engagement is “a positive, fulfilling, work-related state of mind characterized by vigor, dedication, and absorption” [13]. Highly engaged employees seem to be adept at effectively handling negative emotions caused by job burnout [7]. In turn, job burnout can reduce employees’ engaged work behaviors and consequently lead to work inefficiency and low productivity [14,15]. Job satisfaction is “a pleasurable or positive emotional state resulting from the appraisal of one’s job and job experience” [16], involving three aspects: intrinsic, extrinsic, and general [17]. Several academics have also confirmed that job satisfaction is negatively associated with job burnout [18,19,20,21]. Employees with a sense of burnout are usually unsatisfied with the work that they are presently undertaking [22] and have a higher intention of turnover [12].

Job burnout typically occurs among public service workers, such as teachers [6,23,24]. Job burnout not only directly affects teachers’ mental health and job performance [22,25] but also indirectly influences teacher–student relationships and students’ learning outcomes [26]. Prior efforts have revealed the frequency of job burnout in teachers and its deteriorating influence on their perceived organizational support, work engagement, and job satisfaction. For instance, Anomneze et al. [27] argued that perceived organizational support was significantly adversely connected with emotional exhaustion and depersonalization among secondary school teachers in Enugu State, while Xu and Yang [28] illustrated that perceived organizational support was significantly negatively related to all the dimensions of job burnout in teachers at primary and secondary schools in China. Furthermore, Faskhodi [29] revealed a strong negative connection between work engagement and job burnout in teachers teaching English at a private language institute in Tehran, while Hakanen et al. [30] determined that burnout was significantly negatively predictive of work engagement among teachers from elementary, secondary, and vocational schools in Finland. Moreover, Platsidou [25] argued that job dissatisfaction of special teachers in Greece was directly related to their feelings of job burnout, while Skaalvik and Skaalvik [22] highlighted that job satisfaction was closely connected to emotional exhaustion and diminished personal achievement among teachers in Norwegian elementary and middle schools.

In addition, some theoretical and empirical research has acknowledged the interrelationship between job burnout and its correlates. As per social exchange theory (SET) [31,32], perceived organizational support is an essential contributor to job satisfaction. Some scholars have vindicated that employees’ perceptions of organizational support positively predict their levels of job satisfaction [33,34,35]. Moreover, in line with the job demands–resources (JDR) model [36], when employees perceive sufficient job resources (e.g., organizational support), they are inclined to work enthusiastically and with dedication (e.g., work engagement), and are more likely to be satisfied with their work [37,38,39]. Additionally, extant studies have shown that work engagement functions as a mediator in the association between organizational support and job satisfaction [40,41,42].

Existing studies have considerably enhanced our understanding of job burnout and its impacts on individuals’ work-related mental health and well-being. However, these investigators have not yet been incorporated into a synthesized conceptual framework that can recognize organizational and individual factors. Specifically, the interplay between perceived organizational support, work engagement, job satisfaction, and job burnout remains underexplored. More importantly, the cohort of generalist teachers responsible for teaching several disciplines in rural primary schools has not received adequate attention regarding their workload, psychological situation, and career promotion. Compared with their counterparts solely teaching one discipline, a generalist teacher is a more challenging and stressful profession because they have to be able to teach all basic subjects, such as mathematics [43], science [44], music [45], language [46], arts [47], physical education [48], and even dance [49], often to more than one grade level. In addition to broad subject knowledge and skills, they are expected to be equipped with proficient teaching techniques, including information technology, to teach effectively in the classroom. These high requirements may ultimately lead generalist teachers to job dissatisfaction [50] and emotional stress [51], which might cause job burnout, especially for those who work in rural areas with fewer job resources. This situation may be more prevalent in China. Although China has implemented a series of strategic programs, such as the Rural Revitalization Plan and Health China Plan, to promote rural workers’ material and spiritual life quality and their physical and psychological health, rural generalist teachers’ mental health remains unclear. Therefore, there is a great significance in investigating job burnout and its influence on work-related well-being in populations of generalist teachers, which can provide useful empirical insights into equally distributing educational and medical resources to specific groups for policymakers, administrators, and principals around the world.

This study determined the impact of job burnout on perceived organizational support, work engagement, and job satisfaction, using a mediating mechanism. This study provides insights on how job burnout damages employees’ work-related mental health and how perceived organizational support and work engagement cushions the harmful impact of job burnout on job satisfaction in an instance of Chinese generalist teachers in rural primary schools. It can also enrich the literature on job burnout and job satisfaction and contribute to the addition of theoretical knowledge and research paradigms for the JDR model and SET by integrating external and internal factors.

The following hypotheses were formulated:

**Hypothesis** **1** **(H1).** *Job burnout negatively predicts job satisfaction*.

**Hypothesis** **2** **(H2).** *Job burnout negatively predicts perceived organizational support (H2a) and work engagement (H2b)*.

**Hypothesis** **3** **(H3).** *Perceived organizational support positively predicts job satisfaction*.

**Hypothesis** **4** **(H4).** *Work engagement positively predicts job satisfaction*.

**Hypothesis** **5** **(H5).** *Perceived organizational support mediates the connection between job burnout and job satisfaction*.

**Hypothesis** **6** **(H6).** *Work engagement mediates the connection between job burnout and job satisfaction*.

**Hypothesis** **7** **(H7).** *Perceived organizational support and work engagement serially mediate the connection between job burnout and job satisfaction*.

**Figure 1:**
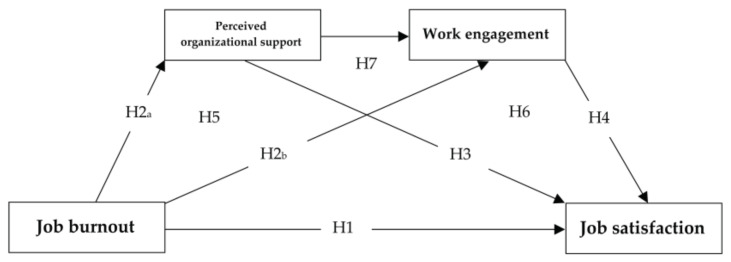
Hypothesized research model.

The complete posited model is presented in Figure 1.

## 2. Materials and Methods

### 2.1. Participants

Participants consisted of 639 generalist teachers working at rural primary schools situated in the rural areas of Guangdong Province in China. Guangdong Province is a microcosm of China’s unbalanced development, whose urban areas, especially in the Pearl River Delta region, are very developed, whereas its rural areas, especially in the eastern, western, and northern regions, are extremely underdeveloped. The underdeveloped economic and educational conditions in rural areas in Guangdong are similar to those in other provinces in China. Therefore, rural generalist teachers’ mental health conditions in Guangdong can reflect the reality of generalist teachers’ well-being in China and strengthen the understanding of the importance of equal access to educational and medical resources for this vulnerable group in a developing country. All participants enrolled in a workshop funded by the local government and were taught by two authors (S.W. and W.C.) to promote their job skills and work performance. Due to the rarity of the participants and to obtain high-quality data, we produced a research method course to enhance their knowledge and skills about how to conduct a study. Through this specific course, their scientific awareness, research skills, and methodological competency, including how to respond to a questionnaire, have been greatly improved. The survey was designed with Wenjuanxing software, and a visiting link was generated and delivered online via the WeChat discussion group attended by the participants from 13 to 17 December 2021; participants could fill it out whenever and wherever. After the participants had fed back about the survey, the data were gathered, and it was determined whether the responses were random or inconsistent. Benefiting from the good preparation, we confirmed that the data obtained from all the participants were highly valid, and ultimately, 639 participants were involved in this study.

### 2.2. Measures

#### 2.2.1. Job Burnout Scale

Job burnout was evaluated using the 22-item Chinese version of the Maslach Burnout Inventory—Human Services Survey [1], consisting of three dimensions: emotional exhaustion, depersonalization, and personal accomplishment. Items were asked on a seven-point Likert-type scale, from 0 (never) to 6 (every day). A sample statement is “I feel burned out from my work.” Higher scores on emotional exhaustion, depersonalization, and diminished personal accomplishment reflected a higher risk of burnout. In the present study, Cronbach’s alpha values for the total items, emotional exhaustion, depersonalization, and personal accomplishment were 0.88, 0.93, 0.91, and 0.90, respectively.

#### 2.2.2. Perceived Organizational Support Scale

Perceived organizational support was calculated using the 8-item Chinese short form of the Perceived Organizational Support Scale [8] extracted by Settoon et al. [52]. Responses were ranked on a five-point Likert-type scale (from 1 = strongly disagree to 5 = strongly agree). A sample statement is “My organization cares about my opinions”. Higher scores represented a higher degree of perceived organizational support. In the current study, the Cronbach’s alpha value was 0.86.

#### 2.2.3. Work Engagement Scale

Work engagement was determined using the 17-item Chinese version of the Utrecht Work Engagement Scale (UWES) [7], consisting of three subscales: vigor, dedication, and absorption. Each item was rated on a seven-point Likert-type scale, from 0 (never) to 6 (always). A sample statement is “At my work, I feel like bursting with energy.” Higher scores meant a higher level of work engagement. In the present study, the Cronbach’s alpha value was 0.95.

#### 2.2.4. Job Satisfaction Scale

Job satisfaction was assessed using the 20-item Chinese version of the Minnesota Satisfaction Questionnaire Short Form [17], comprising three facets: intrinsic (12 items), extrinsic (8 items), and general (20 items). Responses were marked on a five-point Likert-type scale, ranging from 1 (not satisfied) to 5 (extremely satisfied). A sample statement is “The opportunities to work alone.” Higher total scores signified higher satisfaction with work. In this study, the Cronbach’s alpha values for the total, intrinsic, and extrinsic aspects were 0.94, 0.90, and 0.88, respectively.

### 2.3. Data Analysis

We utilized SPSS 25.0 (IBM, New York, NY, USA) and SmartPLS 3.3 (SmartPLS GmbH Company, Oststeinbek, Germany) to analyze the data. We first used Harman’s one-factor test [53] to evaluate the common method variance (CMV). The outcomes indicated that the single-factor extraction alone accounted for 35.23% of the variance, below the 40% requirements. Thus, common method bias did not appear in the present study. The variance inflation factor (VIF) was also employed to examine the multicollinearity issue. The results indicated that all variables did not exceed the cut-off threshold of 5 [54]. Then, descriptive analysis was conducted in SPSS, including means, standard deviations, differences, and correlations. Next, we assessed the psychometric characteristics of the proposed constructs by assessing the measurements’ reliability, convergent validity, and discriminant validity in Smart PLS. To increase the model’s acceptability, we retained the items exceeding the factor loading value of 0.7 for further examination [55]. Consequently, the mediation hypotheses were tested with 5000 bootstrapping subsamples.

### 2.4. Ethics

This study was approved by the South China Normal University Academic Ethics Committee (Reference Number: 20210003). The ethics approval and informed consent form were supplied to the participants before delivering the questionnaire.

## 3. Results

### 3.1. Characteristics of Participants

The participants included 345 women (54%) and 294 men (46%): 8.3% of the participants ranged from 20 to 30 years old; 29.1% from 31 to 40; 49.8% from 41 to 50; and 12.8% were over 50. Years of work experiences spanned from 1 to 5 (8.5%), 6 to 10 (5.0%), 11 to 20 (28.2%), and to over 20 (58.3%) years. Most of the participants (82.2%) had achieved bachelor’s degrees (M.A. = 1.3%, College = 15.2%, and Second vocational school = 1.3%). Participants’ titles of associate professor, senior, primary, and none accounted for 0.2%, 65.5%, 26.2%, and 8.1% of the total, respectively. Most participants (96.4%) signed a long-term contract. Over 90% of the participants worked in public schools (97.8%), compared with private schools (3.6%). More detailed demographical information is provided in Table 1.

### 3.2. Descriptive Statistics

Table 2 showcases the variables’ minimum, maximum, means, and standard deviations. The average job burnout score of generalist teachers was close to the median, indicating that they had experienced moderately work-related burnout, especially fatigued syndrome due to emotional exhaustion. The average perceived organizational support of generalist teachers was slightly higher than the median, meaning that they perceived sufficient support from their schools. Similarly, the average value of work engagement was also greater than the median, especially in dedication, which indicated that the participants had devoted themselves to the workplace. Meanwhile, generalist teachers felt generally satisfied with their occupations, particularly at the intrinsic level, with assets such as self-decidedness, steadiness, and a sense of fulfillment.

Table 3 presents the notable differences between gender, age, experience, and degree. Concerning gender differences, male participants registered significantly higher scores in job burnout (*p* < 0.001). In contrast, female participants scored more highly in perceived organizational support, work engagement, and job satisfaction than males, but only exhibited remarkable distinctions in the two former variables (*p* < 0.05). In terms of age differences, participants aged from 31 to 40 had the highest scores for perceived organizational support, work engagement, and job satisfaction, whereas the youngest participants, between 20 and 30, had the highest scores for job burnout. The differences among these four variables were all significant (*p* < 0.001, 0.001, 0.01, and 0.001, respectively). For experience, generalist teachers who were in their early careers obtained the highest score for job burnout, whereas participants with 6 to 10 years’ work experience scored the highest in perceived organizational support and job satisfaction, and those who had over 20 years’ experience gained the highest scores for work engagement. All the variables yielded statistically significant differences (*p* < 0.001, 0.001, 0.05, and 0.05, respectively). Regarding educational level, participants with master’s degrees scored the highest for all the variables, but significant differences were only identified for job satisfaction (*p* < 0.05). No significant differences were discovered among the other control variables.

### 3.3. Correlation Analysis

Table 4 shows the mutual correlations between the variables and sub-variables and the scales’ Cronbach’s alpha values. All the dimensions were strikingly interconnected with each other. Job burnout and its three components were negatively and significantly associated with the other variables (*p* < 0.01). The correlations were positive and significant between perceived organizational support, work engagement, and job satisfaction (*p* < 0.01). Notably, job satisfaction was strongly associated with perceived organizational support and work engagement (r > 0.5, *p* < 0.01), and moderately related to job burnout and its sub-variables (0.3 < r < 0.5, *p* < 0.01) [56]. The three dimensions of work engagement were strong predictors, and the two dimensions of job satisfaction were also powerfully predictive. However, although all the dimensions of job burnout were strongly related to job burnout, the low personal accomplishment was weakly related to emotional exhaustion and depersonalization. This outcome vindicated the assumption that inefficacy is weakly linked to exhaustion and cynicism [1,6,57].

### 3.4. Measurement Model Test

The measurement model was measured using four parameters: factor loadings, composite reliability (CR), Cronbach’s alpha, and average variance extracted (AVE) [58]. Following MacCallum et al. [59,60], an item factor loading value over 0.60 was considered a feasible solution, and all extracted items met the requirements. The CR and Cronbach’s alpha values should meet the rules of thumb over 0.7, and the AVE threshold should be over 0.5 [61]. As shown in Table 5, Cronbach’s alpha values of constructs varied from 0.85 to 0.95, and the composite reliabilities varied from 0.90 to 0.96. All the AVE values also exceeded the 0.5 requirements. Thereby, the modified outer model displayed appropriate convergent validity.

Discriminant validity was also a crucial criterion for determining the outer model’s adequacy. SmartPLS 3.3 applies the heterotrait–monotrait (HTMT) ratio of construct correlations to test discriminant validity. The results in Table 6 manifested that none of the HTMT ratios between the two latent variables exceeded the suggested value of 0.85 [62]. Therefore, each construct was judged legitimate and divergent from the others.

### 3.5. Structural Model Test

The structural model was evaluated using three parameters: the goodness of fit (GoF) indices, path coefficient, and *t*-value. The GoF criteria consisted of the standardized root-mean-square residual (SRMR) [63], squared Euclidean distance (d_ULS), geodesic distance (d_G) [64], normed fit index (NFI) [65], and root-mean-squared residual covariance (RMS_theta) [66]. The results in Table 7 illustrated that the model fit for the proposed structural model was satisfactory.

**Figure 2:**
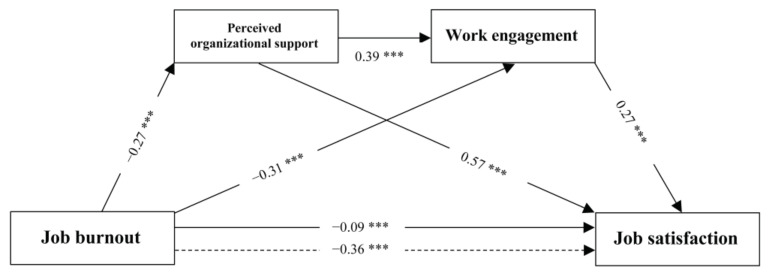
Mediation test. *** *p* < 0.001.

As recommended by Chin [67], the value of the path coefficient should be higher than 0.2, and its related *t*-statistics value should be above 1.96. As depicted in Table 8 and Figure 2, both the total direct effect (*β* = −0.36, *t* = −8.87, *p* = 0.000) and the overall indirect effect (*β* = −0.26, *t* = −8.75, *p* = 0.000) were negatively significant, indicating that the hypothesized construct was statistically valid. 

In the direct paths, job burnout significantly and adversely predicted job satisfaction (*β* = −0.09, *t* = −3.04, *p* = 0.000), fully endorsing Hypothesis 1. Moreover, perceived organizational support (*β* = −0.27, *t* = −6.48, *p* = 0.000) and work engagement (*β* = −0.31, *t* = −8.10, *p* = 0.000) were also negatively predicted by job burnout, totally supporting Hypotheses 2a and 2b. Furthermore, perceived organizational support had a positively significantly predicting effect on job satisfaction (*β* = 0.57, *t* = 18.89, *p* = 0.000). Similarly, work engagement also positively and significantly predicted job satisfaction (*β* = 0.27, *t* = 8.16, *p* = 0.000). Therefore, Hypotheses 3 and 4 were also entirely supported.

In the indirect paths, the total effects were negatively significant for the JB −> POS −> JS path (*β* = −0.15, *t* = −6.21, *p* = 0.000), demonstrating that perceived organizational support mediated the connection between job burnout and job satisfaction. Similarly, the path for JB −> WE −> JS (*β* = −0.09, *t* = −5.70, *p* = 0.000) was also negatively significant, indicating that work engagement also worked as a mediator in the association between job burnout and job satisfaction. Furthermore, the serial path for JB −> POS −> WE −> JS (*β* = −0.03, *t* = −4.77, *p* = 0.000) was also negatively significant, which confirmed the mediating buffering role of perceived organizational support and work engagement in the association between job burnout and job satisfaction. Therefore, Hypotheses 5–7 were proven entirely.

## 4. Discussion

This study assessed the function of perceived organizational support and work engagement as sequential mediators in the link between job burnout and job satisfaction among Chinese generalist teachers in rural primary schools. Participants’ overall averages were above the median, indicating that they were highly cared for by their institutions, effectively engaged in their work tasks, and quite satisfied with their work conditions. However, participants also had a moderate feeling of burnout due to multiple workloads. These findings are aligned with the conclusion that generalist teachers teaching physical education experienced a high level of engagement and enjoyment with their work when perceiving and receiving resource support packages from the organization, such as professional promotion programs [68]. The results also coincide with previous findings indicating that teachers suffered high rates of job burnout [26,30], especially for emotional exhaustion and depersonalization [27]. Therefore, it can be asserted that job burnout is prevalent among teachers across disciplines and cultures.

Furthermore, the difference analysis results revealed that the groups who are male, 20–30 years old, or have 1–5 years of work experience had significantly higher rates of burnout than their counterparts, likened to the empirical findings of Saloviita and Pakarinen [69] that male special teachers expressed higher levels of depersonalization and lower levels of accomplishment than females. These results are also coherent with the results of Antoniou et al. [70] and Brunsting et al. [71] that younger teachers felt more exhausted than their experienced peers. However, these outcomes are incongruent with prior burnout studies that female or older teachers usually encountered a higher risk of exhaustion than males or younger teachers [72,73]. The results are also unlike the findings of Raducu and Stanculescu [74], that gender does not significantly impact teachers’ job burnout. This result indicated that generalist teachers struggling in their early careers were more prone to experiencing burnout. Therefore, more work-related resources and support should be allocated to generalist teachers to facilitate their perceived organizational support and work engagement and ameliorate their job burnout. 

In addition, the results displayed close links between job burnout, perceived organizational support, work engagement, and job satisfaction among Chinese generalist teachers in rural primary schools. The specific interactions between the four investigated variables are detailed in the following subsections.

### 4.1. The effect of Job Burnout on Job Satisfaction

This study found that job burnout had a strongly negative connection with job satisfaction and could significantly and negatively predict job satisfaction. This result indicates that Chinese generalist teachers with higher levels of job burnout have lower degrees of job satisfaction.

This result supports the precedent assumption that job burnout and job dissatisfaction are closely associated [6,25]. Maslach, Schaufeli, and Leiter [6] stated that a drop in job satisfaction could be attributed to an increase in job burnout. When feeling tired of daily work and stressed about effectively accomplishing work targets, employees are at a greater risk of experiencing burnout, which can consequently reduce their satisfaction with work. Conversely, employees with high energy and enjoyment are rarely dissatisfied with their occupation. This study also found that generalist teachers with a low level of job burnout had high job satisfaction. This result also supports the empirical findings of Skaalvik and Skaalvik [22], who highlighted that teachers’ job dissatisfaction was indirectly linked to emotional exhaustion and reduced personal accomplishment. Underlying these findings, it can be legitimated that job burnout is a critical predictor of job satisfaction which should be acknowledged when boosting levels of job satisfaction among generalist teachers. Therefore, stakeholders, such as policymakers and school administrators, should reduce generalist teachers’ work stress by distributing fewer work tasks, empowering more teaching autonomy, or creating closer school contact to decrease their feelings of burnout and ultimately promote their job satisfaction.

### 4.2. The Mediation Role of Perceived Organizational Support

The results showcased that perceived organizational support was negatively connected with job burnout but positively connected with job satisfaction, and could function as a mediator buffering the relationship between job burnout and job satisfaction among Chinese generalist teachers in rural primary schools.

These results are coherent with previous theoretical arguments [8,75]; a lack of organizational support contributes to employees’ job burnout, and its absence can lower the levels of job satisfaction. Consistent with the JDR model [4], the support offered by organizational leaders and fellows can mitigate employees’ feelings of job burnout and promote their job satisfaction [76,77]. When perceiving necessary resources and desirable support, employees can provoke more positive strategies to cope with job demands and subsequently feel less exhausted about repetitive tasks [78]. Conversely, without sufficient organizational support, employees’ feelings of burnout might be enhanced, and their job dissatisfaction might increase [79]. In this study, our data also showed that Chinese generalist teachers perceiving greater organizational support had a lower level of job burnout and a greater level of job satisfaction. These results correspond to the study by Um and Harrison [80], that directors’ support moderates the relationship between job burnout and job dissatisfaction. It is also in line with the finding of Hombrados-Mendieta and Cosano-Rivas [81], that social workers’ perceptions of workplace support as a mediator decreases the negative effect of job burnout on job satisfaction. In addition, our findings support the empirical studies of Molero Jurado et al. [82] and Trinidad [83], that teachers’ perceptions of support in the educational context mediated the impact of job burnout on job satisfaction. Therefore, it can be concluded that perceived organizational support exerts protection against job burnout and is a precursor to job satisfaction.

Based on these findings mentioned above, some targeted initiatives focusing on promoting generalist teachers’ perceived organizational support should be taken to reduce the negative impact of job burnout on job satisfaction. The first feasible strategy is to provide ample material support, such as a high salary, comfortable facilities, and position promotion. Another practicable strategy is to offer desired psychological support, such as value recognition, cultural diversity, and well-being care. When generalist teachers perceive that their contributions are highly appreciated by their affiliated schools, they are more likely to overcome feelings of burnout and heighten their levels of job satisfaction.

### 4.3. The Mediating Role of Work Engagement

This study revealed that work engagement had a significantly negative relationship with job burnout and a positive relationship with job satisfaction; namely, work engagement mediated the association between job burnout and job satisfaction among Chinese generalist teachers in rural primary schools.

The results further confirm the argument that job burnout is opposite to work engagement [6,7]. According to the JDR model, the components of job-related well-being, such as job burnout, work engagement, and job satisfaction, are mutually related. On the one hand, high job demands can induce burnout, which reciprocally results in work disengagement and negative organizational outcomes (e.g., job dissatisfaction). On the other hand, abundant job resources can drive energetic work behavior, which, in turn, reduces mental unhealth (e.g., job burnout) and increases job satisfaction [4,13,36]. Specifically, vigor and dedication of work engagement dimensions are the opposite pool of exhaustion and cynicism of burnout dimensions, respectively. At the same time, work engagement is also the antecedent of job satisfaction [7,84]. In this study, the data also revealed that more engaged generalist teachers had lower levels of job burnout and greater job satisfaction. This outcome also aligns with the empirical results of Hakanen, Bakker, and Schaufeli [30], who reported that teachers’ burnout could negatively predict their work engagement. In addition, Chan et al. [85] summarized that engaged teachers were more satisfied with their work conditions. Therefore, it can be speculated that work engagement has a protective effect against the negative consequence of job burnout and buffer the deteriorating impact of job burnout on job satisfaction among Chinese generalist teachers.

Underpinning these findings, some insightful suggestions concentrating on strengthening generalist teachers’ work engagement might be considered to eliminate the harmful effect of job burnout on job satisfaction. The first possible way is to facilitate teachers’ self-confidence in achieving teaching assignments. According to Bandura’s self-efficacy theory [86], once teachers believe in effectively accomplishing their work tasks, they will positively engage in their work role with energy and vigor. Another informative strategy is to provide professional mentoring and colleague-based guidance, such as training teaching skills and building inexperienced–experienced teaching teams. These measures will be beneficial to generalist teachers for motivating their work attitude and organizational commitment, which, in turn, prevents them from job burnout and fosters their job satisfaction.

### 4.4. The Serial Mediating Role of Perceived Organizational Support and Work Engagement

The results also indicated that perceived organizational support mediated the relationship between job burnout and work engagement, and that work engagement mediated the association between perceived organizational support and job satisfaction; i.e., there was an occurrence of a serial mediation of perceived organizational support and work engagement in the relationship between job burnout and job satisfaction.

These results are familiar with the SET [31,32], which proposes that employees who perceive support from supervisors and peers tend to reciprocatively embed into their work and develop adherence to their organization, resulting in satisfaction with their occupation. In contrast, feeling unsupported might spark disengagement in work behavior and dissatisfaction with the job [79]. This result is also likened to the practical findings which documented that feeling assisted by the organization in autonomy, competency, and relatedness can mobilize generalist teachers to engage in the subject and satisfy the work situation [68,87]. More importantly, the results provide solid evidence to the JDR model through the sequential mediating role of perceived organizational support and work engagement in the association between job burnout and job satisfaction. As posited by Orgambide and colleagues [88,89], work engagement mediates the link between job resources (e.g., leader support) and organizational outcomes (e.g., job satisfaction). At the same time, job resources (e.g., organizational support) also protect employees from deteriorating job demands (e.g., job burnout), motivate employees’ work productivity, and consequently increase their levels of job satisfaction through a mediation process [35,84,90]. Therefore, it is rational that the negative impact of job burnout on job satisfaction can be gradually lowered through the chain mediating path of perceived organizational support and work engagement among Chinese generalist teachers.

Based on these findings, the importance of job burnout interventions is to provide a variety of organizational support, because limited job resources are directly linked to job burnout and indirectly related to work disengagement [30]. Chinese generalist teachers who perceive receiving more organizational support are prone to performing with more engagement and are more inclined to be satisfied with work and feel less exhausted confronting heavy workloads. In addition to the supportive strategies mentioned above regarding the institutional and individual levels, the national policy support is also essential for generalist teachers to boost their work-related well-being, such as implementing the rural generalist teacher cultivation program in several appointed universities to train high-level talents who can adapt to the educational context in the rural areas.

## 5. Limitations and Future Directions

This study undoubtedly has limitations. Firstly, the current study utilized a self-reporting questionnaire to collect data, which might raise issues of social desirability bias. Therefore, despite the common psychological syndrome among Chinese generalist teachers, the causality should be treated with caution. Future studies should adopt additional strategies to avoid this bias, such as inserting bogus items or adding a simple self-reporting honest item. Secondly, this study applied a convenience sampling method that recruited respondents in a specific workshop in one developed province, which might weaken the sample’s representativeness and restrict the generalizability of the findings. Therefore, future studies should extend the sample size by covering diverse regions and schools, especially in underdeveloped areas, to obtain more statistically robust results. Additionally, because the data were collected during the COVID-19 pandemic, the environmental stress resulting from social lockdown and school closure may have further deteriorated participants’ work-related well-being. Therefore, they might have chosen a more negative option than their actual feelings when answering the questionnaire. Therefore, future studies could conduct longitudinal research to compare the results with this study and ultimately determine their real mental health conditions.

## 6. Conclusions

Drawing on the JDR model and SET, this study investigated the sequential mediating role of perceived organizational support and work engagement in the relationship between job burnout and job satisfaction among Chinese generalist teachers in rural primary schools. The results revealed that job burnout negatively predicted job satisfaction, perceived organizational support, and work engagement, whereas perceived organizational support, work engagement, and job satisfaction were positively related. The results also demonstrated that perceived organizational support as a mediator buffered the negative impact of job burnout on job satisfaction and work engagement. Similarly, work engagement also acted as a mediator, protecting the negative effect of job burnout on job satisfaction, and mediating the positive link between perceived organizational support and job satisfaction. Therefore, the serial mediation model of perceived organizational support and work engagement on the association between job burnout and job satisfaction in Chinese generalist teachers in rural primary schools was entirely validated. These empirical results enrich the current literature on individual work-related well-being in the specific populations of generalist teachers in the school context by integrating organizational and individual factors into the JDR model and SET. This study also provides insights into how to offer generalist teachers the diverse and dynamic support they require, promote their engagement at work, accelerate their satisfaction with their job, and ultimately prevent their burnout by giving them more opportunities to enhance their professional competency and offering more flexible psychological guidance. This study could be further improved by optimizing the research design and enlarging the sample size and diversity to enhance the generalization of the results.

## Figures and Tables

**Table 1:** Participants’ characteristics.

Characteristics	Sub−Characteristics	Number	Percentage
Gender	Male	294	46.0%
	Female	345	54.0%
Age	20–30	53	8.3%
	31–40	186	29.1%
	41–50	318	49.8%
	Over 50	82	12.8%
Experience	1–5 years	54	8.5%
	6–10 years	32	5.0%
	11–20 years	180	28.2%
	Over 20 years	373	58.3%
Degree	Master’s	8	1.3%
	Bachelor’s	526	82.2%
	College	97	15.2%
	Second vocational school	8	1.3%
Title	Associate professor	1	0.2%
	Senior	419	65.5%
	Junior	167	26.2%
	None	52	8.1%
Affiliation	Public	625	97.8%
	Private	14	2.2%
Contract	Long-term	616	96.4%
	Fixed-term	23	3.6%

**Table 2:** Minimum, maximum, mean, and standard deviation of variables and sub-variables.

Variables	Minimum	Maximum	Average	Standard Deviation
Job burnout	0.60	5.87	2.22	0.98
Emotional exhaustion	1.00	7.00	3.13	1.34
Depersonalization	1.00	7.00	2.00	1.22
Low personal accomplishment	0.00	6.00	1.60	1.41
Perceived organizational support	1.25	5.00	3.63	0.59
Work engagement	2.00	7.00	5.39	0.92
Activity	2.00	7.00	5.36	0.94
Dedication	2.00	7.00	5.68	1.05
Absorption	2.00	7.00	5.18	1.05
Job satisfaction	2.15	5.00	3.71	0.51
Intrinsic satisfaction	2.33	5.00	3.80	0.51
Extrinsic satisfaction	1.63	5.00	3.58	0.58

**Table 3:** Significant differences in gender, age, experience, and degree.

Variables	Gender						Age (Years)										Experience (Years)										Degree									
	Male (*n* = 294)		Female (*n* = 345)		*t*	*Sig.*	20–30 (*n* = 53)		31–40 (*n* = 186)		41–50 (*n* = 318)		50 above (*n* = 82)		*F*	*Sig.*	1–5 (*n* = 54)		6–10 (*n* = 32)		11–20 (*n* = 180)		20 above (*n* = 373)		*F*	*Sig.*	Master (*n* = 8)		Bachelor (*n* = 526)		College (*n* = 97)		SVS (*n* = 8)		*F*	*Sig.*
	*M*	*SD*	*M*	*SD*			*M*	*SD*	*M*	*SD*	*M*	*SD*	*M*	*SD*			*M*	*SD*	*M*	*M*	*SD*	*M*	*SD*	*M*			*M*	*SD*	*M*	*SD*	*M*	*SD*	*M*	*SD*		
JB	2.37	1.01	2.08	0.93	3.73	0.00 ***	2.91	0.88	2.17	0.98	2.13	0.94	2.21	1.00	10.46	0.00 ***	2.85	0.94	2.34	0.91	2.16	0.96	2.14	0.96	8.88	0.00 ***	2.58	1.39	2.20	0.96	2.22	1.04	2.57	0.77	0.76	0.52
POS	3.57	0.61	3.69	0.57	−2.50	0.01 *	3.52	0.57	3.78	0.63	3.61	0.55	3.46	0.59	7.01	0.00 ***	3.50	0.60	3.82	0.63	3.71	0.60	3.60	0.57	3.63	0.00 ***	3.86	0.60	3.65	0.58	3.52	0.64	3.39	0.82	2.31	0.08
WE	5.29	0.93	5.48	0.91	−2.57	0.01 *	4.84	0.73	5.44	0.92	5.44	0.93	5.43	0.92	7.01	0.00 **	4.87	0.78	5.35	0.77	5.41	0.98	5.45	0.91	6.42	0.02 *	5.73	1.20	5.39	0.90	5.37	1.00	4.93	1.05	1.02	0.39
JS	3.71	0.53	3.72	0.48	−0.35	0.73	3.48	0.52	3.77	0.53	3.73	0.48	3.69	0.51	4.50	0.00 ***	3.51	0.55	3.76	0.48	3.72	0.53	3.74	0.48	3.30	0.01 *	4.14	0.69	3.72	0.49	3.68	0.56	3.38	0.57	3.29	0.02 *

* *p* < 0.05, ** *p* < 0.01, *** *p* < 0.001; JB: job burnout, POS: perceived organizational support, WE: work engagement, JS: job satisfaction, SVS: secondary vocational school, n: number, M: mean, SD: standard deviation.

**Table 4:** Bivariate correlations.

Variables	1	2	3	4	5	6	7	8	9	10	11	12
1. Job burnout	(0.88)											
2. Emotional Exhaustion	0.73 **	(0.90)										
3. Depersonalization	0.77 **	0.65 **	(0.91)									
4. Low personal accomplishment	0.71 **	0.11 **	0.24 **	(0.90)								
5. Perceived organizational support	−0.35 **	−0.28 **	−0.29 **	−0.22 **	(0.85)							
6. Work engagement	−0.49 **	−0.29 **	−0.30 **	−0.45 **	0.38 **	(0.95)						
7. Activity	−0.44 **	−0.28 **	−0.25 **	−0.40 **	0.37 **	0.92 **	(0.88)					
8. Dedication	−0.49 **	−0.29 **	−0.36 **	−0.41 **	0.41 **	0.90 **	0.76 **	(0.91)				
9. Absorption	−0.43 **	−0.23 **	−0.24 **	−0.42 **	0.27 **	0.92 **	0.76 **	0.74 **	(0.90)			
10. Job satisfaction	−0.46 **	−0.33 **	−0.32 **	−0.35 **	0.63 **	0.65 **	0.58 **	0.61 **	0.59 **	(0.94)		
11. Intrinsic satisfaction	−0.48 **	−0.30 **	−0.31 **	−0.41 **	0.56 **	0.68 **	0.61 **	0.63 **	0.63 **	0.96 **	(0.91)	
12. Extrinsic satisfaction	−0.38 **	−0.33 **	−0.29 **	−0.24 **	0.64 **	0.52 **	0.45 **	0.51 **	0.45 **	0.92 **	0.77 **	(0.88)

** *p* < 0.01; Cronbach’s alpha values are listed in parentheses diagonally. The numbers 1 to 12 in the first line refer to the variables with the same digits in the first column.

**Table 5:** Results of measurement model test.

Constructs	Items	Factor Loading	Cronbach	Composite Reliability	Average Variance Extracted
Job burnout	Emotional exhaustion 5	0.80	0.85	0.90	0.64
	Depersonalization 6	0.87			
	Depersonalization 7	0.88			
	Depersonalization 8	0.82			
	Lack of Personal Accomplishment 13	0.61			
Perceived organizational support	Perceived organizational support 1	0.83	0.87	0.90	0.65
	Perceived organizational support 2	0.84			
	Perceived organizational support 3	0.84			
	Perceived organizational support 6	0.76			
	Perceived organizational support 8	0.78			
Work engagement	Activity 1	0.74	0.95	0.96	0.59
	Activity 2	0.79			
	Activity 3	0.76			
	Activity 5	0.71			
	Dedication 7	0.82			
	Dedication 8	0.83			
	Dedication 9	0.83			
	Dedication 10	0.81			
	Dedication 11	0.71			
	Absorption 12	0.76			
	Absorption 13	0.73			
	Absorption 14	0.75			
	Absorption 15	0.83			
	Absorption 16	0.72			
	Absorption 17	0.72			
Job satisfaction	Intrinsic 11	0.75	0.92	0.93	0.63
	Intrinsic 15	0.80			
	Intrinsic 16	0.80			
	Intrinsic 20	0.77			
	Extrinsic 5	0.81			
	Extrinsic 6	0.79			
	Extrinsic 12	0.84			
	Extrinsic 19	0.78			

**Table 6:** Heterotrait–monotrait (HTMT) discrimination validity.

Variables	1	2	3
1. Job burnout	−		
2. Perceived organizational support	0.30	−	
3. Work engagement	0.43	0.51	−
4. Job satisfaction	0.39	0.81	0.62

**Table 7:** Fit indices of the structural model.

Fit Index	SRMR	d_ULS	d_G	NFI	RMS_theta
Proposed value	<0.10	>0.05	>0.05	>0.80	<0.12
Estimated value	0.07	2.54	0.72	0.83	0.11

SRMR: standardized root-mean-square residual, d_ULS: squared Euclidean distance, d_G: geodesic distance, NFI: normed fit index, RMS_theta: root-mean-squared residual covariance.

**Table 8:** Results of the hypothesis test.

Hypotheses	Path	*β*	*t*	Decision
H1	JB −> JS	−0.09	–3.04 ***	Supported
H2a	JB → POS	−0.27	–6.48 ***	Supported
H2b	JB → WE	−0.31	–8.10 ***	Supported
H3	POS → JS	0.57	18.89 ***	Supported
H4	WE → JS	0.27	8.16 ***	Supported
H5	JB → POS → JS	−0.15	–6.21 ***	Supported
H6	JB → WE → JS	−0.09	–5.70 ***	Supported
H7	JB → POS → WE −> JS	−0.03	–4.77 ***	Supported
Total effect		−0.36	–8.87 ***	Supported
Total indirect effect		−0.26	–8.75 ***	Supported

*** *p* < 0.001; POS: perceived organizational support, WE: work engagement, JS: job satisfaction, JB: job burnout.

## Data Availability

The data supporting the results of this study are available from the corresponding author upon reasonable request.
